# The chinese version of achilles tendon total rupture score: cross-cultural adaptation, reliability and validity

**DOI:** 10.1186/s12955-016-0574-8

**Published:** 2017-01-05

**Authors:** Jin Cui, Zhenyu Jia, Xin Zhi, Xiaoqun Li, Xiao Zhai, Liehu Cao, Weizong Weng, Jun Zhang, Lin Wang, Xiao Chen, Jiacan Su

**Affiliations:** 1 Graduate Management Unit, Changhai hospital affiliated to the Second Military Medical University, Shanghai, People’s Republic of China; 2 Department of Orthopedics, Changhai hospital affiliated to the Second Military Medical University, Shanghai, People’s Republic of China

**Keywords:** Achilles tendon rupture, ATRS, Cross-cultural, Chinese, Reliability, Validity

## Abstract

**Background:**

The Achilles tendon Total Rupture Score (ATRS), which is originally developed in 2007 in Swedish, is the only patient-reported outcome measure (PROM) for specific outcome assessment of an Achilles tendon rupture.Purpose of this study is to translate and cross-culturally adapt Achilles tendon Total Rupture Score (ATRS) into simplified Chinese, and primarily evaluate the responsiveness, reliability and validity.

**Methods:**

International recognized guideline which was designed by Beaton was followed to make the translation of ATRS from English into simplified Chinese version (CH-ATRS). A prospective cohort study was carried out for the cross-cultural adaptation. There were 112 participants included into the study. Psychometric properties including floor and ceiling effects, Cronbach’s alpha, intraclass correlation coefficient, effect size, standard response mean, and construct validity were tested.

**Results:**

The mean scores of CH-ATRS are 57.42 ± 13.70. No sign of floor or ceiling effect was found of CH-ATRS. High level of internal consistency was supported by the value of Cronbach’s alpha (0.893). ICC (0.979, 95%CI: 0.984-0.993) was high to indicate the high test-retest reliability. Great responsive ness was proved with the high absolute value of ES and SRM (0.84 and 8.98, respectively). The total CH-ATRS score had very good correlation with physical function and body pain subscales of SF-36 (r = −0.758 and r = −0.694, respectively, *p* < 0.001), while poor correlation with vitality and role physical subscales of SF-36 (r = −0.033 and r = −0.025, respectively, *p* ≥ 0.05), which supported construct validity of CH-ATRS.

**Conclusion:**

This Chinese version of Achilles tendon Total Rupture Score (CH-ATRS) can be used as a reliable and valid instrument for Achilles tendon rupture assessing in Chinese-speaking population.

*Level of evidence II*

**Electronic supplementary material:**

The online version of this article (doi:10.1186/s12955-016-0574-8) contains supplementary material, which is available to authorized users.

## Background

The Achilles tendon rupture (ATR) is the most common tendon rupture disease in the human body [21], and the risk factors includes running, jumping, and sudden acceleration or deceleration [15, 23]. The incidence of Achilles tendon rupture is up to 18 per 100,000 per year and is still increasing [16]. ATR causes pain, muscle strength reduction, functional ability affection and daily activity limitation to the patients [18, 19, 33].

There are several clinical tests to diagnose Achilles tendon rupture, including the Simmonds or Thompson’s test, the calf squeeze test, and the palpation of the gap test on tendon body, however the exact symptoms and disabilities caused by ATR cannot be reflected [4, 9, 19]. The Achilles tendon Total Rupture Score (ATRS), which is originally developed in 2007 in Swedish, is the only patient-reported outcome measure (PROM) for specific outcome assessment of an Achilles tendon rupture [24].

The ATRS is short, simple, and easy to use as PROM. Before being used in different language and culture groups, the ATRS should not only be translated, but also be adapted to the local culture. And, the translation and adaptation should follow the cross-cultural adaptation guidelines described by Beaton and Guillemin [4, 10]. Currently, the ATRS has been translated and cross-cultural validated to several languages, including English [5], Swedish [24], Danish [8], Turkish [13], Persian [2], and Italian [30]. There is no reliable and valid Simplified Chinese version of ATRS yet.

We hypothesized that the Simplified Chinese version of ATRS (CH-ATRS) would be a reliable and valid instrument to evaluate the Achilles tendon rupture in China after the translation and cross-cultural adaptation process. The purpose of our study is to perform a cross-cultural adaptation and translation of the original version of ATRS into Simplified Chinese and evaluate the validity, responsiveness and reliability of the Simplified Chinese version.

## Methods

### Translation and cross-cultural adaptation

**Fig. 1:**
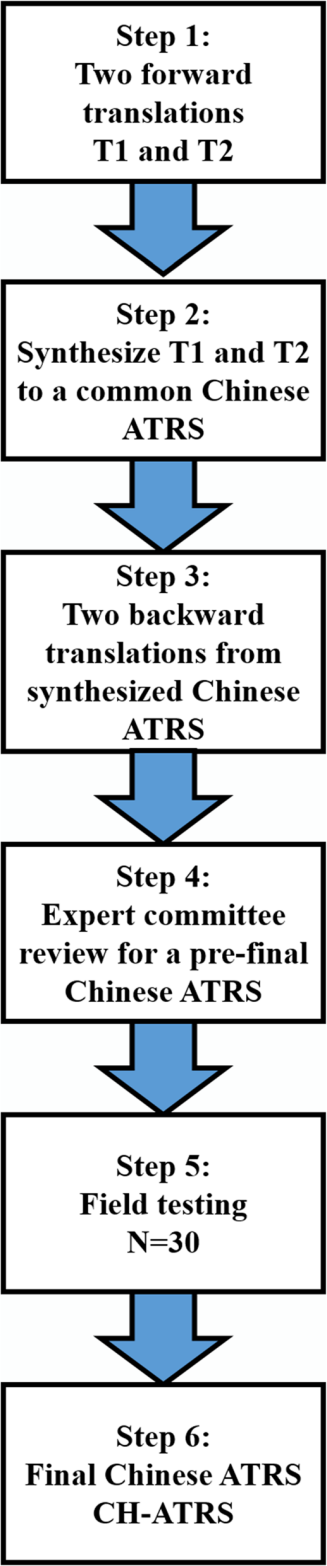
Flow chart of translation and cross-cultural adaptation of the Achilles tendon Total Rupture Score (ATRS) to Chinese language

The translation and cross-cultural adaptation of ATRS was in accordance with the guideline designed by Beaton and Guillemin, which is also recommended of the American Academy of Orthopedic Surgeons (AAOS) outcome committee [3]. Although the ATRS was developed based on Swedish population, it was published in English language [24]. The translation process including the following 3 steps: Step 1, two translators were responsible for the original literal and conceptual translation of the ATRS. Of the two translators, the informed one was an orthopedic surgeon of our department, and the uninformed was a full-time translator with no medical background. Step 1 was ended by independent complete of the two translators. Step 2, according to consensus of two initial translators and an expert committee, a common Chinese ATRS was synthesized. Step 3, another two bilingual translators whose first language was English back-translated the synthesized Chinese ATRS to English to highlight conceptual errors in the translations. Step 4, according to the consensus of the four translators and an expert committee, a pre-final version of Chinese ATRS was approved. Step 5, thirty patients participated in the final comprehension test of the pre-final version to complete the final version of Chinese ATRS. The total procedure to complete the translation and adaptation to Chines is shown in Fig. 1.

### Participants and data collection

The sample size was determined according to the quality criteria described by Terwee et al. that the study should enroll in at least 100 patients for internal consistency analysis and 50 patients for floor or ceiling effects, reliability, and validity analysis [28].

The inclusion criteria were as follows: age of 18 and older, ability to speak Chinese Mandarin and read Simplified Chinese, reference to acute ATR and to be treated with surgical therapy. The exclusion criteria were as follows: patients with other lower limb injury which could affect lower extremities’ functions, patients with bilateral rupture, and patients with physical therapy related to Achilles tendon in the previous one month and patients who had bad compliance. Patient of different ages, social, ethnic and educational background were included.

All the participants signed informed consents, and this study was approved by the clinical research ethics committee of Changhai hospital (NO. CHEC2015-011).

At the first time of the data collection, all of the included patients completed the demographic data, CH-ATRS, and the Short Form 36 (SF-36). A second-time data collection were finished seven days after the first visit to clinic, to evaluate the test-retest reliability of CH-ATRS. And a third-time data collection were finished six months later after surgery and proper rehabilitation for responsiveness evaluation.

### Instruments

The outcome measures used in this study were the translated version of ATRS (CH-ATRS) and a validated Chinese version of the SF-36.

The ATRS is a ten-item questionnaire to evaluate symptoms and physical activity in patients with Achilles tendon rupture. For each question of the questionnaire, patients are asked to respond using an 11-grade Likert scale by checking a box labelled 0–10. A maximal score of 100 indicates no symptoms and full function, whereas a minimum score of 0 indicates severe symptom and no function [24].

SF-36 is a widely used instrument, which consists of 36 questions on the general health status of patients [32], with eight health concept subscales including, physical functioning (PF), bodily pain (BP), general health (GH), vitality (VT), social functioning (SF), role-physical (RP), role-emotional (RE) and mental health (MH). SF-36 has also been translated and culturally adapted into Chinese [17].

### Psychometric assessments and statistical analysis

The analyses were performed in SPSS for windows Release 21.0 (Chicago, IL). A *p* value of less than 0.05 was considered statistically significant for all analyses. The percentage of missing data of less than 5% was considered acceptable.

### Ceiling and floor effects

The term ceiling and floor effects, which present if the lowest score or highest score on one question was greater than 15%, was analysed [28].

### Reliability

The term reliability of CH-ATRS refers to repeatability or consistency, which is divided into two major categories: internal consistency and reproducibility or test-retest reliability. Internal consistency is evaluated with the Cronbach’s alpha, and the coefficient was also calculated for elimination of 1 item in all 12 questions. The value of Cronbach’s alpha between 0.70 and 0.95 indicated a good internal consistency [28] All items were examined for correlation with the overall score [26, 29].

The second test was done 7 days after the first test. Test-retest reliability was assessed with intraclass correlation coefficient (ICC) and the Bland-Altman plot. The result of ICC evaluation was divided into 5 categories, including excellent (>0.8), good (0.61-0.80), moderate (0.41-0.60), fair (0.21-0.40) and poor (≤0.20) [1]. The Bland-Altman plot could be used to measure within-subject variation and limits of agreement [25].

### Responsiveness

Responsiveness was assessed by comparing the results of first and third test of CH-ATRS, with calculating the standard response mean (SRM) and the effect size (ES).

Values of SRM were considered large (SRM ≥ 0.80), moderate (SRM = 0.50-0.79), and small (SRM = 0.20-0.49). Values of ES of 0.20, 0.50, and 0.80 or greater have been proposed to represent small, moderate, and large responsiveness, respectively [12].

### Validity

Construct validity was calculated by the Pearson’s correlation coefficient (r) of CH-ATRS with the SF-36. Correlations were categorized as follows: poor (0–0.20), fair (0.21-0.40), moderate (0.41-0.60), very good (0.61-0.80), or excellent (0.8-1.0) [7]. It was hypothesized that CH-ATRS was strongly correlated with the PF and BP subscales of the SF-36, moderate with the GH, RP and SF subscales of SF-36, and poorly correlated with the mental health related subscales of the SF-36.

## Results

### Translation and cultural adaptation

During forward and back-translation of ATRS, there were no major problem or large language difficulty existed. And no major problem was revealed during the cross-cultural adaptation. Small revisions were made to ensure better comprehension for native Chinse-speaking population. For the proper noun Achilles tendon was replaced with traditional Chinese word “Genjian”, which is the routine Chinese expression of Achilles tendon. And the final version could represent the original version in China.

### Descriptive statistics

Altogether 112 patients were recruited in the study (Table 1). The 1st-Test was conducted at the beginning of this research (112 patients), the 2nd-Test was conducted one week later to calculate the test-retest reliability (ICC) of the CH-ATRS (112patients), and the 3rd-Test was conducted six months later to calculate the responsiveness (ES, SRM) of the CH-ATRS (91 patients). Of 112 patients 104 (92.8%) are male and 8 (7.2%) are female, with the mean age of 44.5 ± 9.7 years old. Most of the patients had been educated in universities, with the mean education time of 13.0 ± 4.3 years. For the involved side, 50 (44.6%) had ATR with right side.

**Table 1:** Demographic and clinical characteristics of participants

Characteristics	Number or Mean ± SD
Total number of patients	112
Age (Year)	
Mean ± SD	44.5 ± 9.7
Gender	
Male (%)	104 (92.8)
Female (%)	8 (7.2)
Involved side	
Right (%)	50 (44.6)
Left (%)	62 (55.4)
BMI	23.3 ± 4.5

*SD* standard deviation

### Floor and ceiling effects

The distribution of the CH-ATRS scores is good, which ranged from 18 to 94 (Table 2). No floor or ceiling effects were observed. No patient was scored the highest or lowest score in test or retest. There was no data missed during the whole test.

**Table 2:** Score distribution of CH-ATRS

Scale	No. of Items	Mean ± SD	Observed range	Floor effect (%)*	Ceiling effect (%)^a^
CH-ATRS	10	57.42 ± 13.70	18-94	0.00	0.00

*CH-ATRS*, Chinese version of Achilles tendon Total Rupture Score

^a^Percentage of patients with the worst (floor effect) and the best (ceiling effect) condition

### Reliability

The Cronbach’s alpha of the total questionnaire for internal consistency evaluation of CH-ATRS was 0.893 (Table 3), which proved the internal consistency of CH-ATRS was good. And all items correlated with the total score and elimination of one item, all 10 items did not result in an alpha less than 0.871 (Table 3).

**Table 3:** Internal consistency of CH-ATRS

Question	Mean ± SD if item deleted	Corrected item-total correlation	Alpha if item removed
1	51.90 ± 12.14	0.762	0.879
2	52.48 ± 12.25	0.698	0.885
3	52.45 ± 12.17	0.742	0.881
4	52.90 ± 12.33	0.642	0.891
5	52.46 ± 12.36	0.665	0.890
6	52.84 ± 12.56	0.616	0.892
7	50.42 ± 12.66	0.793	0.879
8	50.34 ± 12.61	0.769	0.879
9	50.43 ± 12.50	0.950	0.871
10	50.54 ± 12.44	0.788	0.877
Total score	57.42 ± 13.70	1.000	0.893

*SD* standard deviation

**Fig. 2:**
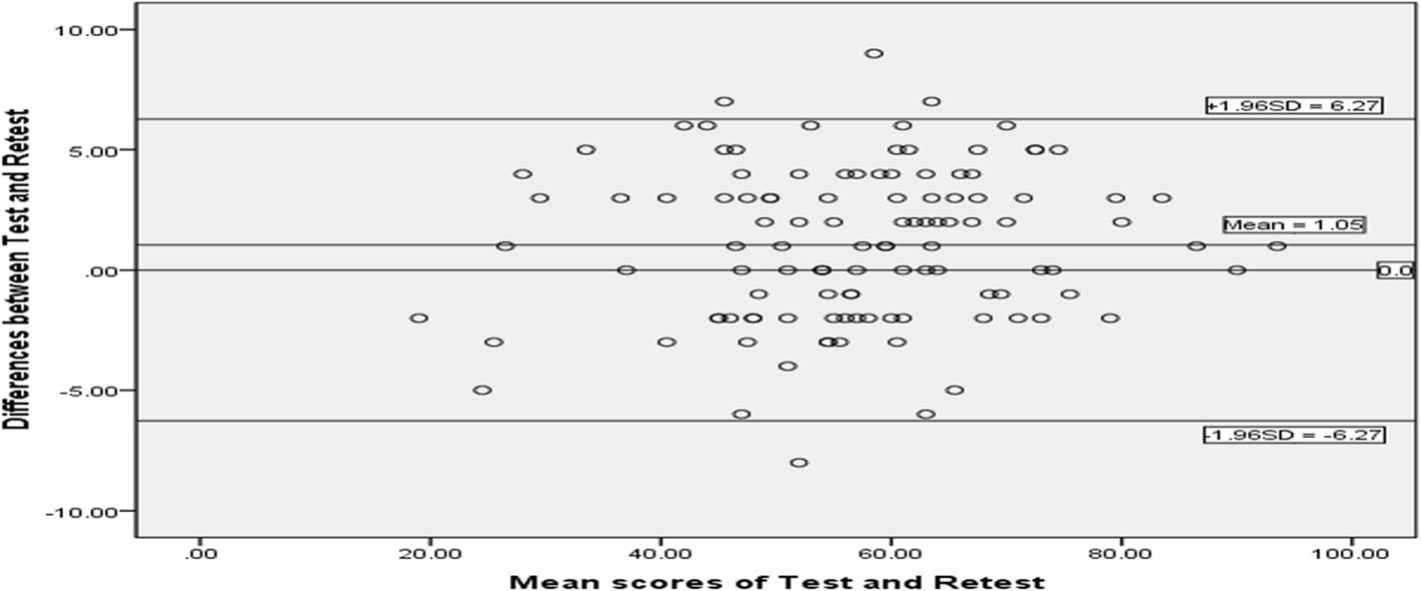
Bland-Altman plot showing differences between Test and Retest

The mean ± SD of the total scale was 57.42 ± 13.70 (56.55 ± 13.27, the 2nd time). The ICC for total score was 0.986 (95%CI: 0.980-0.990) (Table 4), which indicated excellent test-retest reliability. There was no systematic bias between the test and retest evaluation of all scores according to the Blant-Altman plot (Fig. 2).

**Table 4:** Test-retest reliability and responsiveness of the CH-ATRS

Scale	1st-Test (mean ± SD)	2nd-Test (mean ± SD)	3rd-Test (mean ± SD)	ICC (95%CI)	ES	SRM
CH-ATRS	57.42 ± 13.70	56.55 ± 13.27	42.74 ± 13.66	0.986 (0.980-0.990)	−1.01	−4.81

*ICC* intra-class correlation coefficient, *ES* effect size, *SRM* standardized response mean, *CI* 95% confidence interval, *CH-ATRS* Chinese version of Shoulder Pain and Disability Index

The 1st-Test was conducted at the beginning of this research (112 patients), the 2nd-Test was conducted one week later to calculate the test-retest reliability (ICC) of the CH-ATRS (112 patients), and the 3rd-Test was conducted six months later to calculate the responsiveness (ES, SRM) of the CH-ATRS (91 patients)

### Responsiveness

The responsiveness of CH-ATRS was showed to be great, as the absolutely values of ES and SRM were 1.01 and 4.81.

### Validity

CH-ATRS had very good correlation with the PF and BP subscales of SF-36 (*r* = −0.758 and −0.694, respectively), moderate correlation with RP, GH, and SF subscales of SF-36 (*r* = −0.470, −0.537 and −0.510, respectively), fair correlation with the MH subscales of SF-36 (*r* = −0.219), and poor correlation with the VT and RE subscales of SF-36 (r = −0.033 and −0.025, respectively) (Table 5).

**Table 5:** Constructive validity of the CH-ATRS

Correlation coefficient r	CH-ATRS
SF-36 subscales	
Physical Function, PF	‘-0.758**
Role Physical, RP	’-0.470**
Bodily Pain, BP	‘-0.694**
General Health, GH	’-0.537**
Vitality, VT	‘-0.033
Social Function, SF	‘-0.510**
Role Emotional, RE	’-0.025
Mental Health, MH	‘-0.219**

**: *p* < 0.001

*CH-ATRS* Chinese version of Achilles tendon Total Rupture Score, *SF-36* Short Form 36

## Discussion

In this study, the English version of ATRS was successfully translated and adapted into Chinese. Statistical analysis indicated that CH-ATRS was reliable and valid, and the CH-ATRS can be used in Chinese population to evaluate the clinical condition after Achilles tendon rupture. There was no major problem and missing data during the process of adaptation and evaluation, which indicated good acceptance of CH-ATRS. After the adaptation, CH-ATRS was supported to be a feasible instrument for Chinese with ATR.

There are several instruments to evaluate quality of life (QoL) for patients with foot and ankle injuries, including VISA-A (the Victorian Institute of Sports Assessment Achilles questionnaire) [20], FAOS (the Foot and Ankle Outcome Score) [27], and AOFAS (American Orthopaedic Foot and Ankle Society) [10], etc. AOFAS and FAOS have been used for Achilles tendon rupture [10, 31], which were not developed for Achilles tendon pathologies specifically [8], and none of these questionnaire is translated and cross-cultural adapted into Chinese. SF-36 has been adapted into Chinese for the evaluation of general health status of patients [11]. The correlation with subscales of SF-36 was used for the evaluation of the constructive validity of CH-ATRS.

The existence of floor or ceiling effects may result in the overestimation of agreement parameters [6]. As the results of our study showed (Table 2), there was no floor or ceiling effect of CH-ATRS, which indicated that CH-ATRS questionnaire, can be used to measure the change in prospective studies. The former studies also reported no floor or ceiling effect of the cross-culturally adapted version of ATRS in English [14], Danish [8], Turkish [13], and Persian [2], The floor and ceiling effects were not reported for the adapted version in Italian language [30].

The reliability of CH-ATRS was proved to be good, according to the results of Cronbach’s alpha (Table 3) and ICC (Table 4). The value of Cronbach’s alpha of the total score (0.893) indicated a high level of internal consistency. And the stability of Cronbach’s alpha when each item was deleted indicated the high level of correlation and balance among each item. The value of ICC (0.986, 95%CI: 0.980-0.990) indicated the excellent test-retest reliability of CH-ATRS. According to the former study, the recommended intervals for test-retest evaluation range from 2 days to 2 weeks [22], and we choose 7 days for this study. The test-retest internal 7 days ensured that no change in the ATR status and probably no memory-based response. The results for evaluation of CH-ATRS are similar with those of the other versions of ATRS [2, 8, 13, 30].

The ES and SRM values (Table 4) indicated great responsiveness of CH-ATRS. Responsiveness is an important measurement property of a questionnaire for evaluation of different types of treatments. The absolute value of ES and SRM of 0.80 or larger represent great responsiveness [12]. The values of the original Swish version are 0.87 and 2.21, respectively of ES and SRM [24].

The construct validity was evaluated with the correlation coefficient between CH-ATRS and SF-36, which is the commonly used instrument in China. SF-36 is a common used questionnaire for evaluation of QoL of patients. SF-36 and the simplified version SF-12 are commonly used to evaluate the construct validity of different versions of ATRS [8, 13, 30]. As the results showed, CH-ATRS had very good correlation with the physical functioning and body pain subscales of SF-36, moderate correlation with role physical, general health, and social function subscales of SF-36, fair and poor correlation with mental health, vitality, and role emotional subscales of SF-36. As ATRS is a specific instrument for evaluation of pain, symptom, and function disability resulted from ATR, it's reasonable to see high level of correlation with physical function and body pain subscale, and low level of correlation with mental health, vitality, and role emotional subscales. It is also similar with the result of cross-cultural adaptation of other versions of ATRS [8, 13, 30].

There are two limitations in our study. First, this is a single-centre research, of which the patients were all from one hospital and may not fully represent the whole population who speak Chinese. Second, as the specific instrument for evaluation of foot and ankle injury such as FAOS and AOFAS have not been translated and cross-cultural adapted into Chinese, we only used SF-36 for evaluation of construct validity.

## Conclusion

This study supports that the Chinese version of Achilles tendon Total Rupture Score (CH-ATRS) can be used as a reliable and valid instrument for Achilles tendon rupture assessing in Chinese-speaking population.

## Additional file

Additional file 1: Questionnaire score details of the whole study. (PDF 302 kb)

## Abbreviations

AAOS: The American Academy of Orthopedic Surgeons
AOFAS: American Orthopaedic Foot and Ankle Society
ATRS: Achilles tendon total rupture score
CH-ATRS: Chinese version of ATRS
ES: The effect size
FAOS: The foot and ankle outcome score
ICC: Intraclass correlation coefficient
PROM: Patient-reported outcome measure
SRM: The standard response mean
VISA-A: The Victorian Institute of Sports Assessment Achilles questionnaire

## References

[1] Altman DG, Schulz KF, Moher D, Egger M, Davidoff F, Elbourne D, Gotzsche PC, Lang T, Consort G (2001). The revised CONSORT statement for reporting randomized trials: explanation and elaboration. Ann Intern Med.

[2] Ansari NN, Naghdi S, Hasanvand S, Fakhari Z, Kordi R, Nilsson-Helander K (2016). Cross-cultural adaptation and validation of Persian Achilles tendon Total Rupture Score. Knee surgery, sports traumatology, arthroscopy.

[3] Beaton DE, Bombardier C, Guillemin F, Ferraz MB (2000). Guidelines for the process of cross-cultural adaptation of self-report measures. Spine (Phila Pa 1976).

[4] Button G, Pinney S (2004). A meta-analysis of outcome rating scales in foot and ankle surgery: is there a valid, reliable, and responsive system?. Foot Ankle Int.

[5] Carmont MR, Silbernagel KG, Nilsson-Helander K, Mei-Dan O, Karlsson J, Maffulli N (2013). Cross cultural adaptation of the Achilles tendon Total Rupture Score with reliability, validity and responsiveness evaluation. Knee Surg Sports Traumatol Arthrosc.

[6] Ekeberg OM, Bautz-Holter E, Tveita EK, Keller A, Juel NG, Brox JI (2008). Agreement, reliability and validity in 3 shoulder questionnaires in patients with rotator cuff disease. BMC Musculoskelet Disord.

[7] Feise RJ, Michael Menke J (2001). Functional rating index: a new valid and reliable instrument to measure the magnitude of clinical change in spinal conditions. Spine (Phila Pa 1976).

[8] Ganestam A, Barfod K, Klit J, Troelsen A (2013). Validity and reliability of the Achilles tendon total rupture score. J Foot Ankle Surg.

[9] Garras DN, Raikin SM, Bhat SB, Taweel N, Karanjia H (2012). MRI is unnecessary for diagnosing acute Achilles tendon ruptures: clinical diagnostic criteria. Clin Orthop Relat Res.

[10] Guillemin F, Bombardier C, Beaton D (1993). Cross-cultural adaptation of healthrelated quality of life measures: literature review and proposed guidelines. J Clin Epidemiol.

[11] Hamido F, Al Harran H, Al Misfer AR, El Khadrawe T, Morsy MG, Talaat A, Elias A, Nagi A (2015). Augmented short undersized hamstring tendon graft with LARS(R) artificial ligament versus four-strand hamstring tendon in anterior cruciate ligament reconstruction: preliminary results. Orthop Traumatol Surg Res.

[12] Husted JA, Cook RJ, Farewell VT, Gladman DD (2000). Methods for assessing responsiveness: a critical review and recommendations. J Clin Epidemiol.

[13] Kaya Mutlu E, Celik D, Kilicoglu O, Ozdincler AR, Nilsson-Helander K (2015). The Turkish version of the Achilles tendon Total Rupture Score: cross-cultural adaptation, reliability and validity. Knee Surg Sports Traumatol Arthrosc.

[14] Kearney RS, Achten J, Lamb SE, Parsons N, Costa ML (2012). The Achilles tendon total rupture score: a study of responsiveness, internal consistency and convergent validity on patients with acute Achilles tendon ruptures. Health Qual Life Outcomes.

[15] Leppilahti J, Puranen J, Orava S (1996). Incidence of Achilles tendon rupture. Acta Orthop Scand.

[16] Leppilahti J, Orava S (1998). Total Achilles tendon rupture. A review. Sports Med.

[17] Li L, Wang HM, Shen Y (2003). Chinese SF-36 Health Survey: translation, cultural adaptation, validation, and normalisation. J Epidemiol Community Health.

[18] Maffulli N (1998). The clinical diagnosis of subcutaneous tear of the Achilles tendon. A prospective study in 174 patients. Am J Sports Med.

[19] Maffulli N, Ajis A (2008). Management of chronic ruptures of the Achilles tendon. J Bone Joint Surgery Am Vol.

[20] Maffulli N, Longo UG, Testa V, Oliva F, Capasso G, Denaro V (2008). Italian translation of the VISA-A score for tendinopathy of the main body of the Achilles tendon. Disabil Rehabil.

[21] Maffulli N, Waterston SW, Squair J, Reaper J, Douglas AS (1999). Changing incidence of Achilles tendon rupture in Scotland: a 15-year study. Clin J Sport Med.

[22] Marx RG, Menezes A, Horovitz L, Jones EC, Warren RF (2003). A comparison of two time intervals for test-retest reliability of health status instruments. J Clin Epidemiol.

[23] Movin T, Ryberg A, McBride DJ, Maffulli N (2005). Acute rupture of the Achilles tendon. Foot Ankle Clin.

[24] Nilsson-Helander K, Thomee R, Silbernagel KG, Thomee P, Faxen E, Eriksson BI, Karlsson J (2007). The Achilles tendon Total Rupture Score (ATRS): development and validation. Am J Sports Med.

[25] Olofsen E, Dahan A, Borsboom G, Drummond G (2015). Improvements in the application and reporting of advanced Bland-Altman methods of comparison. J Clin Monit Comput.

[26] Roddey TS, Olson SL, Cook KF, Gartsman GM, Hanten W (2000). Comparison of the University of California-Los Angeles Shoulder Scale and the Simple Shoulder Test with the shoulder pain and disability index: single-administration reliability and validity. Phys Ther.

[27] Roos EM, Brandsson S, Karlsson J (2001). Validation of the foot and ankle outcome score for ankle ligament reconstruction. Foot Ankle Int.

[28] Terwee CB, Bot SD, de Boer MR, van der Windt DA, Knol DL, Dekker J, Bouter LM, de Vet HC (2007). Quality criteria were proposed for measurement properties of health status questionnaires. J Clin Epidemiol.

[29] Tugay U, Tugay N, Gelecek N, Ozkan M (2011). Oxford Shoulder Score: cross-cultural adaptation and validation of the Turkish version. Arch Orthop Trauma Surg.

[30] Vascellari A, Spennacchio P, Combi A, Grassi A, Patella S, Bisicchia S, Canata GL, Zaffagnini S, Committee SS Cross-cultural adaptation and multi-centric validation of the Italian version of the Achilles tendon Total Rupture Score (ATRS). Knee surgery, sports traumatology, arthroscopy. 2016 1–8 10.1007/s00167-016-4152-8 27139231

[31] Venditto T, Tognolo L, Rizzo RS, Iannuccelli C, Di Sante L, Trevisan M, Maggiolini FR, Santilli V, Ioppolo F (2015). 17-Italian Foot Function Index with numerical rating scale: development, reliability, and validity of a modified version of the original Foot Function Index. Foot.

[32] Ware JE, Sherbourne CD (1992). The MOS 36-item short-form health survey (SF-36). I. Conceptual framework and item selection. Medical Care.

[33] Zhao HM, Yu GR, Yang YF, Zhou JQ, Aubeeluck A (2011). Outcomes and complications of operative versus non-operative treatment of acute Achilles tendon rupture: a meta-analysis. Chin Med J.

