# Supplementary material for: The chinese version of achilles tendon total rupture score: cross-cultural adaptation, reliability and validity
Source: Health Qual Life Outcomes. 2017 Jan 5;15:2. doi: 10.1186/s12955-016-0574-8 (PMC5220618; doi:10.1186/s12955-016-0574-8)
Supplement: Additional file 1: — Questionnaire score details of the whole study. (PDF 302 kb) [file 12955_2016_574_MOESM1_ESM.pdf]

No.	ATRS									
	1	2	3	4	5	6	7	8	9	10
1	8	8	8	7	7	7	9	9	9	9
2	8	2	7	7	2	3	6	7	7	7
3	7	6	5	5	5	6	6	7	7	8
4	8	8	8	5	0	7	8	8	8	8
5	1	9	8	0	7	1	6	6	6	7
6	5	5	6	3	3	7	7	6	7	6
7	5	6	5	5	5	6	8	9	8	6
8	6	6	6	6	6	5	8	8	8	8
9	4	4	5	4	4	4	6	7	6	6
10	8	7	4	1	8	4	7	7	7	7
11	5	5	5	3	5	5	7	6	7	8
12	5	4	7	6	5	2	7	9	7	6
13	2	6	2	4	2	6	7	8	6	4
14	4	5	4	1	7	5	7	7	7	7
15	6	7	4	5	5	5	7	7	7	7
16	5	6	6	1	5	5	5	6	7	8
17	6	0	7	0	6	3	4	8	6	4
18	8	4	9	5	8	5	9	9	9	9
19	4	4	3	3	3	7	6	6	6	7
20	1	8	5	1	1	7	7	6	6	6
21	5	5	5	6	5	2	7	8	7	7
22	1	1	1	1	2	2	6	4	5	4
23	4	3	3	3	5	1	7	9	6	3
24	5	5	4	5	2	5	7	7	7	7
25	6	6	4	6	2	6	7	7	6	6
26	4	4	4	4	4	3	6	6	6	6
27	10	10	9	10	9	4	9	9	10	10
28	7	3	8	4	8	6	8	8	8	7
29	9	5	8	5	9	4	8	8	8	8
30	5	6	5	5	6	6	7	7	8	8
31	6	6	6	6	6	5	8	7	8	7
32	7	5	3	3	7	5	7	6	7	7
33	7	8	1	6	7	3	6	7	7	7
34	5	3	7	3	5	4	8	7	7	5
35	2	2	1	1	0	1	5	4	4	4
36	7	7	5	4	5	5	8	7	7	6
37	8	1	7	0	1	2	3	8	6	3
38	7	4	5	5	5	6	8	8	7	8
39	8	8	5	4	4	5	7	8	8	7
40	7	7	7	6	5	7	9	9	9	9
41	6	6	6	6	6	6	8	8	8	8
42	4	4	4	4	4	4	8	8	5	2
43	8	8	8	9	7	9	9	9	9	9
44	2	2	2	3	2	1	6	4	5	4
45	6	6	5	7	6	7	9	8	8	7
46	6	6	6	5	5	5	6	8	7	6
47	7	6	5	5	5	6	7	8	7	8
48	4	4	2	0	7	4	4	5	5	7
49	0	5	2	7	7	0	7	5	6	6
50	4	3	4	4	2	6	9	8	6	3
51	6	6	6	7	6	6	8	7	8	9

52	5	5	3	7	2	4	7	6	6	5
53	4	4	5	5	1	7	6	6	6	7
54	4	3	3	5	3	3	6	6	6	6
55	5	1	6	1	1	8	6	7	6	7
56	8	4	8	5	9	5	9	9	9	9
57	2	2	4	5	5	5	8	3	6	9
58	8	8	2	8	8	2	8	8	8	7
59	1	2	2	2	6	2	5	6	5	6
60	6	6	6	6	6	6	8	9	8	8
61	1	0	1	0	0	1	4	4	4	3
62	7	5	9	5	8	4	9	8	9	9
63	6	5	3	8	2	5	7	5	7	8
64	4	4	4	4	7	1	7	6	6	5
65	7	7	5	4	6	5	5	8	7	8
66	7	7	6	6	5	5	9	9	9	8
67	5	5	4	5	2	5	6	7	7	7
68	7	7	5	4	5	5	6	8	8	8
69	3	5	5	5	5	4	7	6	7	7
70	8	7	5	4	5	4	7	8	7	8
71	4	4	7	1	4	1	8	4	6	8
72	5	5	1	1	6	2	6	6	6	8
73	5	3	3	1	5	4	6	7	6	7
74	7	7	7	4	8	6	9	9	9	8
75	8	8	9	8	9	8	9	9	9	10
76	9	9	10	9	9	9	10	10	10	9
77	6	3	4	4	7	4	7	7	7	7
78	2	2	3	3	5	2	5	5	5	4
79	8	9	5	5	7	3	7	6	7	7
80	7	7	7	6	5	8	8	8	8	9
81	8	4	9	5	8	7	9	9	9	9
82	8	2	4	5	6	2	7	6	7	6
83	8	0	1	9	1	8	6	6	6	6
84	6	1	3	7	4	2	6	6	6	6
85	4	3	2	3	2	4	4	5	5	6
86	6	6	5	6	6	5	7	8	8	8
87	4	4	4	7	4	3	6	7	7	8
88	4	4	4	4	4	5	6	6	6	6
89	5	5	5	6	5	5	7	6	7	7
90	5	5	5	6	6	7	7	8	7	8
91	6	6	6	6	6	5	8	9	8	8
92	7	4	8	4	8	4	9	9	9	8
93	5	5	4	5	2	6	7	7	7	6
94	7	6	5	5	5	6	8	8	7	5
95	9	5	8	4	8	4	9	8	9	9
96	4	1	7	7	1	7	5	7	7	7
97	7	1	8	8	1	1	6	5	6	6
98	4	4	2	3	3	7	6	4	5	6
99	7	7	5	4	4	5	7	6	8	8
100	6	7	4	8	4	8	9	8	8	8
101	6	6	5	5	6	6	8	8	8	8
102	6	5	4	7	4	2	7	6	7	8
103	6	5	6	5	5	6	8	7	8	8
104	4	4	3	3	3	7	6	6	7	8

105	1	1	1	1	1	1	5	4	4	3
106	8	8	5	6	3	3	8	5	7	7
107	7	7	9	6	7	9	9	9	9	9
108	5	5	5	6	6	4	7	6	7	8
109	9	8	4	5	3	4	7	6	6	7
110	5	5	6	6	6	6	8	8	8	7
111	6	7	7	9	7	8	8	9	8	9
112	2	2	3	2	0	0	5	6	5	5
					ATRS 2					
1	9	9	9	7	7	7	8	8	8	7
2	6	7	7	3	3	7	8	2	7	7
3	7	7	8	6	6	3	7	6	5	5
4	8	8	8	7	7	2	8	8	8	5
5	6	6	7	4	5	1	4	9	8	0
6	7	7	6	7	6	5	5	5	6	3
7	8	8	6	6	6	3	5	6	5	5
8	8	8	8	5	5	6	6	6	6	6
9	6	6	6	4	4	5	4	4	5	4
10	6	7	7	4	7	9	8	7	4	7
11	8	7	8	5	7	4	5	5	5	3
12	7	7	6	2	5	5	5	4	7	6
13	3	6	4	6	3	5	5	6	4	4
14	8	7	7	5	3	9	4	5	4	4
15	7	7	7	5	4	7	6	7	4	5
16	8	7	8	5	4	4	5	6	6	1
17	8	6	4	3	3	1	6	4	7	4
18	8	9	9	5	7	6	8	4	9	5
19	6	6	7	7	2	4	4	4	3	3
20	6	6	6	7	8	1	1	8	5	1
21	7	7	7	5	5	5	5	5	5	6
22	5	5	4	2	3	3	1	1	1	1
23	3	6	3	5	6	8	4	3	9	3
24	6	7	7	5	6	4	5	5	4	5
25	6	6	6	6	1	3	6	6	4	6
26	6	6	6	3	6	6	4	4	4	4
27	10	10	10	4	8	9	10	10	9	10
28	8	8	7	6	3	6	7	3	8	4
29	9	8	8	4	8	8	9	7	8	5
30	8	8	8	6	6	6	5	6	5	5
31	8	8	7	5	5	4	6	6	6	6
32	7	7	7	5	5	4	7	5	6	6
33	6	7	7	3	2	8	7	8	1	6
34	7	7	5	4	4	5	5	3	7	3
35	3	4	4	1	5	1	2	2	4	1
36	7	7	6	5	3	6	7	7	5	4
37	9	6	3	2	0	1	8	6	7	0
38	6	7	8	6	6	9	7	9	5	5
39	9	8	7	5	3	6	8	8	5	4
40	10	9	9	7	7	7	7	7	7	6
41	8	8	8	6	6	6	6	6	6	6
42	4	5	7	4	2	3	4	4	4	4
43	9	9	9	9	6	7	8	8	8	9
44	5	5	4	1	2	2	2	2	2	3

45	9	8	7	7	7	6	6	6	7	7
46	8	7	6	5	6	7	6	6	6	5
47	6	7	8	6	6	3	7	6	5	5
48	5	5	7	4	4	4	4	4	2	0
49	5	6	6	0	4	4	0	5	2	7
50	3	6	3	6	1	8	4	3	4	4
51	8	8	9	6	4	6	6	6	6	7
52	7	6	5	4	5	5	5	5	3	7
53	6	6	7	7	2	5	4	4	5	5
54	6	6	6	3	8	3	4	3	3	5
55	6	6	7	8	5	6	5	6	6	1
56	9	9	9	5	9	4	8	4	8	5
57	4	6	9	5	4	3	2	2	4	5
58	8	8	7	2	8	8	8	8	4	8
59	4	5	6	2	3	3	8	2	2	2
60	8	8	8	6	5	6	6	6	6	6
61	4	4	3	1	1	1	1	4	1	0
62	9	9	9	4	8	8	7	5	9	5
63	7	7	8	5	4	5	6	5	3	8
64	7	6	5	1	7	1	4	4	4	4
65	7	7	8	5	3	6	7	7	5	4
66	9	9	8	5	6	6	7	7	6	6
67	8	7	7	5	6	4	5	5	4	5
68	8	8	8	5	3	6	7	7	5	4
69	7	7	7	4	7	7	3	5	5	5
70	5	7	8	4	2	4	8	7	5	4
71	2	6	8	1	6	5	4	4	7	6
72	6	6	8	2	8	4	5	5	4	1
73	6	6	7	4	5	4	5	3	3	1
74	9	9	8	6	9	5	7	7	7	7
75	9	9	10	8	8	9	8	8	9	8
76	10	10	9	9	9	9	9	9	10	9
77	7	7	7	4	2	9	6	3	4	4
78	5	5	4	2	3	2	2	2	3	3
79	8	7	7	3	3	7	8	9	5	5
80	8	8	9	8	4	6	7	7	7	6
81	9	9	9	7	6	6	8	4	9	5
82	8	7	6	2	6	3	8	2	4	5
83	7	6	6	8	0	3	8	0	1	9
84	6	6	6	2	8	2	6	1	3	7
85	5	5	6	4	2	1	4	3	2	3
86	7	8	8	5	5	6	6	6	5	6
87	7	7	8	3	8	3	4	4	4	7
88	6	6	6	5	2	4	4	4	4	4
89	7	7	7	5	5	5	5	5	5	6
90	6	7	8	7	4	5	5	5	5	6
91	8	8	8	5	5	6	6	6	6	6
92	8	9	8	4	8	5	7	4	8	4
93	7	7	6	6	4	5	5	5	4	5
94	8	7	5	6	6	3	7	6	5	5
95	8	9	9	4	8	3	9	5	8	4
96	7	7	7	7	6	3	4	1	7	7
97	6	6	6	1	9	1	7	1	8	8
98	5	5	6	7	6	4	4	4	2	3

99	8	8	8	5	3	6	7	7	5	4
100	9	8	8	8	5	9	6	7	4	8
101	8	8	8	6	6	6	6	6	5	5
102	7	7	8	2	3	5	6	5	4	7
103	8	8	8	6	6	6	6	5	6	5
104	6	7	8	7	2	4	4	4	3	3
105	5	4	3	1	1	2	1	4	1	5
106	8	7	7	3	1	6	8	8	5	6
107	9	9	9	9	6	7	7	7	9	6
108	7	7	8	7	7	5	5	5	5	6
109	6	6	7	4	3	9	9	8	4	5
110	8	8	7	6	6	6	5	5	6	6
111	8	8	9	8	9	9	6	7	7	9
112	5	5	5	0	1	1	2	2	3	2

ATRS 3

1	8	5	8	6	6	6	7	7	7	6
2	5	6	4	2	2	6	7	1	6	6
3	6	6	7	3	5	2	6	5	4	4
4	7	7	7	6	4	1	7	7	7	4
5	5	5	6	0	4	0	0	8	7	0
6	6	6	5	6	5	2	4	4	5	2
7	7	7	5	5	5	2	2	5	4	4
8	7	7	7	4	4	5	5	3	5	5
9	5	5	5	3	0	4	3	3	2	3
10	5	6	6	3	6	2	7	6	3	0
11	7	6	7	4	6	3	4	4	4	0
12	6	6	5	1	4	4	4	3	4	5
13	2	5	3	5	2	4	1	3	1	3
14	7	6	6	4	2	6	3	4	3	0
15	6	6	6	2	3	6	5	6	3	4
16	7	4	5	4	3	3	4	5	5	0
17	7	5	3	2	2	0	5	0	6	0
18	7	6	8	4	6	5	7	3	8	4
19	3	5	6	6	1	3	3	3	2	2
20	5	5	5	3	5	0	0	7	4	0
21	6	6	6	1	4	4	4	4	4	5
22	4	2	3	1	2	2	0	0	0	0
23	2	3	2	0	5	1	3	2	2	2
24	5	6	4	4	5	3	4	4	3	4
25	5	5	5	3	0	2	5	5	3	5
26	5	5	5	2	3	0	3	3	3	3
27	9	9	9	3	7	8	9	9	8	9
28	7	7	6	5	2	5	6	2	7	3
29	8	7	7	3	5	3	8	4	7	4
30	7	5	5	5	5	5	4	5	4	4
31	7	7	6	4	4	3	5	5	3	5
32	6	6	6	4	4	3	4	4	2	2
33	5	6	4	2	1	7	6	7	0	5
34	6	4	4	3	3	4	4	2	6	2
35	2	3	3	0	0	0	1	1	0	0
36	6	6	5	4	2	5	4	6	4	3
37	8	5	2	1	0	0	5	0	6	0
38	5	6	5	5	5	2	6	3	4	4
39	8	7	4	4	2	5	7	7	4	3

40	9	8	6	6	6	6	4	6	6	5
41	7	5	5	5	5	5	5	5	5	5
42	3	4	1	3	1	2	3	3	3	3
43	8	6	8	8	5	6	7	7	7	8
44	4	2	3	0	1	1	1	1	1	2
45	6	7	6	6	6	5	5	5	4	6
46	7	6	5	4	5	4	5	5	5	4
47	5	4	5	5	5	2	6	5	4	4
48	4	4	4	3	3	3	3	3	1	0
49	4	5	3	0	3	3	0	4	1	6
50	2	5	2	5	0	7	3	2	3	3
51	7	7	8	5	3	5	5	5	5	4
52	6	5	4	3	4	4	4	4	2	4
53	5	5	6	4	1	4	3	3	4	4
54	5	5	3	2	7	2	3	2	2	4
55	5	5	4	7	4	5	4	0	5	0
56	8	8	6	4	8	3	7	3	7	4
57	3	5	6	4	3	2	1	1	3	4
58	7	7	4	1	7	7	7	7	1	7
59	3	4	3	1	2	2	0	1	1	1
60	7	7	5	5	4	5	5	5	5	5
61	3	3	2	0	0	0	0	0	0	0
62	8	8	6	3	7	5	6	4	8	4
63	6	6	5	4	3	4	5	4	2	7
64	6	5	4	0	6	0	3	3	3	3
65	6	4	7	4	2	5	6	6	4	3
66	8	6	7	4	5	5	6	6	5	5
67	5	6	6	4	5	3	4	4	3	4
68	5	7	7	4	2	5	6	6	4	3
69	4	6	6	3	6	3	2	4	4	4
70	4	6	5	3	1	3	7	6	4	3
71	1	5	5	0	5	4	3	3	6	0
72	5	5	5	1	7	3	4	4	0	0
73	5	5	4	3	4	3	4	2	2	0
74	8	8	5	5	8	4	6	6	6	3
75	8	8	7	7	7	6	7	7	8	7
76	9	9	8	8	8	6	6	8	9	8
77	6	6	4	3	1	6	5	2	3	3
78	4	4	3	1	2	1	1	1	2	2
79	7	6	4	2	2	6	7	8	4	4
80	7	7	6	7	3	5	6	6	6	5
81	8	6	6	6	5	5	7	3	8	4
82	7	6	5	1	5	2	5	1	3	4
83	6	5	5	5	0	2	5	0	0	8
84	5	3	5	1	7	1	5	0	2	6
85	4	4	3	3	1	0	3	2	1	2
86	6	5	5	4	4	5	5	5	4	5
87	6	4	5	2	7	2	3	3	3	6
88	3	5	5	4	1	3	3	3	3	3
89	6	4	4	4	4	4	4	4	4	5
90	5	6	5	4	3	4	4	4	4	5
91	7	5	5	4	4	5	5	5	5	5
